# Five Circular RNAs in Metabolism Pathways Related to Prostate Cancer

**DOI:** 10.3389/fgene.2021.636419

**Published:** 2021-01-26

**Authors:** Lili Zhang, Wei Zhang, Hexin Li, Xiaokun Tang, Siyuan Xu, Meng Wu, Li Wan, Fei Su, Yaqun Zhang

**Affiliations:** ^1^Clinical Biobank, Beijing Hospital, National Center of Gerontology, National Health Commission, Institute of Geriatric Medicine, Chinese Academy of Medical Sciences, Beijing, China; ^2^Department of Pathology, Beijing Hospital, National Center of Gerontology, National Health Commission, Institute of Geriatric Medicine, Chinese Academy of Medical Sciences, Beijing, China; ^3^Department of Urology, Beijing Hospital, National Center of Gerontology, National Health Commission, Institute of Geriatric Medicine, Chinese Academy of Medical Sciences, Beijing, China; ^4^The Key Laboratory of Geriatrics, Beijing Institute of Geriatrics, Beijing Hospital, National Center of Gerontology, National Health Commission, Institute of Geriatric Medicine, Chinese Academy of Medical Sciences, Beijing, China

**Keywords:** prostate cancer, circular RNA, miRNA-mRNA, pathways, bioinformatics

## Abstract

Prostate cancer (PCa) is the most common malignant tumor in men, and its incidence increases with age. Serum prostate-specific antigen and tissue biopsy remain the standard for diagnosis of suspected PCa. However, these clinical indicators may lead to aggressive overtreatment in patients who have been treated sufficiently with active surveillance. Circular RNAs (circRNAs) have been recently recognized as a new type of regulatory RNA that is not easily degraded by RNases and other exonucleases because of their covalent closed cyclic structure. Thus, we utilized high-throughput sequencing data and bioinformatics analysis to identify specifically expressed circRNAs in PCa and filtered out five specific circRNAs for further analysis—hsa_circ_0006410, hsa_circ_0003970, hsa_circ_0006754, hsa_circ_0005848, and a novel circRNA, hsa_circ_AKAP7. We constructed a circRNA-miRNA regulatory network and used miRNA and differentially expressed mRNA interactions to predict the function of the selected circRNAs. Furthermore, survival analysis of their cognate genes and PCR verification of these five circRNAs revealed that they are closely related to well-known PCa pathways such as the MAPK signaling pathway, P53 pathway, androgen receptor signaling pathway, cell cycle, hormone-mediated signaling pathway, and cellular lipid metabolic process. By understanding the related metabolism of circRNAs, these circRNAs could act as metabolic biomarkers, and monitoring their levels could help diagnose PCa. Meanwhile, the exact regulatory mechanism for AR-related regulation in PCa is still unclear. The circRNAs we found can provide new solutions for research in this field.

## Introduction

Prostate cancer (PCa) is a slow-growing malignant tumor, the incidence of which increases with age (Carroll and Mohler, 2018; Etzioni and Nyame, 2020). At the beginning of diagnosis, most patients are asymptomatic; however, it is still among the top three causes of cancer-related deaths in men (Siegel et al., 2017). Patients with a high risk of PCa must undergo periodic testing for serum prostate-specific antigen (PSA). Tissue biopsy remains the care standard for diagnosis for suspected PCa (Litwin and Tan, 2017). After the tumor is confirmed by biopsy, the next step is to determine the invasiveness of tumor cells. The Gleason score is the most commonly used scale to assess the grade of PCa. When the grade is high, tumors tend to spread. Most Gleason scores used to evaluate prostate biopsy samples range from 6 to 10. A score of 6 indicates low-risk PCa; a score of 7 indicates intermediate-risk PCa; a score of 8 to 10 indicates high-risk PCa (2019). However, researchers have found that the “normal” PSA level of 0–4 ng/mL does not guarantee cancer-free status; in approximately 25% of men with a PSA below 4 ng/mL, a biopsy still reveals PCa (Kitagawa et al., 2014). Thus, these clinical indexes cannot guarantee the reliability of diagnosis. New assistant biomarkers need to be developed for the diagnosis of PCa.

With the development of sequencing technology, circRNAs are recognized as a new type of regulatory RNA. They were first identified by analysis using next generation RNA sequencing (RNA-seq) in a study of pediatric acute lymphoblastic leukemia (Salzman et al., 2012). Most circRNAs are composed of protein-coding exons; thus, the expression of these circRNAs competes with the production of pre-mRNAs. These events also lead to the expression of circRNAs being higher than that of their cognate linear RNAs under certain conditions (Jeck et al., 2013; Jeck and Sharpless, 2014). CircRNAs can function by directly regulating gene expression or by acting as miRNA sponges (Tay et al., 2014). They are similar to competitive endogenous RNAs (ceRNAs) and contain miRNA response elements (MREs). Therefore, they can function by competing with mRNAs to bind miRNAs (Hansen et al., 2013). For example, dysregulation of circRNA-0001946 contributes to tumor cell proliferation and metastasis in colorectal cancer by targeting microRNA-135a-5p (Deng et al., 2020). CircRNAs are not easily degraded by RNases and other exonucleases due to a covalent closed cyclic structure without free 5′ or 3′ends. They have a longer half-life (>48 h) than linear RNAs (Suzuki et al., 2006; Jeck and Sharpless, 2014). CircRNAs have been known to be rich in tumors (Salzman et al., 2012). Therefore, compared with other RNAs, circRNAs have more advantages as novel biomarkers of cancer and other diseases (Arnaiz et al., 2019).

Studies have shown that circRNAs are functional in PCa. Overexpression of circ0005276 and its host gene X-linked inhibitor of apoptosis protein (XIAP) can promote cell proliferation, migration, and epithelial–mesenchymal transition in PCa tissues compared with that in normal tissues (Feng et al., 2019). CircRNAs can act as oncogenes in the progression of PCa and are differentially expressed between cancer tissues and normal tissues (Feng et al., 2019). It is also reported that circRNAs can act as therapeutic targets. For example, the overexpression of circRNA cir-ITCH significantly inhibits the proliferation, migration, and invasion of PCa cells. By targeting miR-17 in PC-3 and LNCaP cell lines, circRNAs could act as therapeutic targets in PCa, especially in castration-resistant prostate cancer (CRPC) (Li et al., 2020). CircRNAs also affect carbohydrate, lipid, and amino acid metabolism in cancer. By regulating transcription factors, circRNAs can modulate glycolysis (Yu et al., 2019). Thus, it is important to identify differentially expressed circRNAs in PCa and explore their potential as diagnostic and therapeutic targets in cancer.

In this study, we compared circRNAs between four PCa tissues and two adjacent normal tissues of two PCa patients by sequencing six sets of RNA-seq. We selected five circRNAs that were highly expressed in tumor tissues and found that the fold change in expression of these five circRNAs was significantly higher than that of their cognate linear RNAs. We verified these circRNAs by PCR in PCa cell lines and used the circRNA-miRNA-mRNA method to predict biological pathways regulated by these circRNAs. Some well-known pathways in PCa were enriched, such as the p53 signaling pathway, MAPK signaling pathway, hormone-mediated signaling pathway, and cellular lipid metabolic process. These pathways also confirmed the high reliability of the five circRNAs that participated in the regulation of PCa.

## Materials and Methods

### Patients and Samples

For sequencing samples, two pairs of PCa tissues and adjacent tissues were derived from surgical samples. Sections from normal and malignant tissues were examined after staining with hematoxylin and eosin. The tumor specimen comprised >80% malignant cells, and the benign specimen comprised an approximately equal admixture of normal epithelial and stromal cells. The pathology of the prostate tumor was checked by a pathologist and established as a combined Gleason Score of 6 (3 + 3), stage T2a, with focal involvement of the surgical margin. RNA was purified from minced frozen tissue using Trizol reagent (Life Technologies, Inc., Rockville, MD, United States). Total RNA was briefly treated with DNase I. For each sample, an RNA library was constructed using 3 μg total RNA. Ribo-Zero Gold Kits were used to remove rRNA. According to the instructions of the NEB-Next Ultra Directional RNA Library Prep Kit for Illumina (NEB, Ispawich, United States), different index tags were selected. The constructed libraries were sequenced using Illumina; the sequencing strategy used was PE150.

For qRT-PCR, 20 pairs of PCa tissues and adjacent normal tissues were collected from the Department of Pathology of Beijing Hospital with Gleason scores of 6 (14 cases) and 7 (6 cases). All tissues were fixed in phosphate-buffered formalin, dehydrated with ethanol, and embedded in paraffin. The malignant status and Gleason score were obtained for these samples by histological analysis. The work was approved by the Beijing Hospital Ethics Committee.

None of these patients had undergone hormonal therapy prior to surgery.

### Quality Control and Mapping of Sequencing Data

The quality of the fastq data of RNA-seq was evaluated using fastQC (Andrews, 2010). We found some reads mixed with adapters, and then Trimmomatic (Bolger et al., 2014) was used to filter these sequences. The reads with lengths of less than 28 were dropped. The read quality was filtered through a four-base sliding window with an average quality threshold of 15. After the reads passed the sequence quality tests, the filtered reads were mapped to the human hg38 genome (Lander et al., 2001) using the aligner software STAR (Dobin et al., 2013) with parameter “–chimSegmentMin 10.”

### Differential Expressed circRNAs Filtered

CIRCexplorer2 (Zhang et al., 2016) and CLEAR/CIRCexplorer3 (Ma et al., 2019) were mainly used to obtain circRNAs in our research. For CIRCexplorer2, we used the parse module to analyze circRNA fusion junction reads and annotate modules for circRNA gene information. The expression of circRNAs was quantified by CIRCscore in CLEAR/CIRCexplorer3, which indicates the circRNA expression level by linear RNA expression level adjustment. The expression of fold change between tumor circRNAs and normal adjacent prostatic tissue circRNAs was calculated to filter tumor-specific expressed circRNAs.

### Bioinformatics Analysis of CircRNAs

All of the interaction binding sites between circRNAs and miRNAs were downloaded from circBank (Liu et al., 2019). For the novel circRNAs, we used miRNDB (Chen and Wang, 2020) to predict the related miRNAs. We identified the function of the predicted miRNAs by manual literature mining. Then, Cytoscape (Shannon et al., 2003) was used to build a network between circRNAs and miRNAs. For differentially expressed mRNAs, we chose featureCounts (Liao et al., 2014) to quantify read counts for each gene. Based on paired-end data, “requireBothEndsMapped = TRUE” and “isPairedEnd = TRUE” were set additionally. Then, we calculated the normalized expression levels in fragments per kilobase per million mapped reads (FPKM) by using the DGEList and rpkm function from edgeR (Robinson et al., 2010).

To predict circRNA-related pathways, we regarded miRNAs as a middleman to find circRNA-related mRNAs. The interactions between miRNAs and mRNAs were obtained from miRDB (Chen and Wang, 2020). Meanwhile, it provided interaction scores to assess accuracy; only scores higher than 90 and differentially expressed mRNAs were considered for further analysis.

### Functional Enrichment Analysis and Survival Analysis

The circRNA-related mRNA list was analyzed using the functional enrichment tool GOseq (Young et al., 2010). Compared with other tools, GOseq can alleviate selection bias more effectively. The pathways were drawn using ggplot2 (Wickham, 2016) in the R language. For survival analysis, TCGA PCa (PRAD) data were selected in UCSC Xena (Goldman et al., 2020) with progression free intervals to draw Kaplan-Meier plots of circRNA cognate genes. The expression level of the gene was used for survival analysis.

### RNA Extraction and Real-Time Quantitative PCR (qPCR)

A total of 20 PCa tissues and 20 adjacent normal tissue samples were prepared. RNA was extracted from three 10-μm FPE sections per sample. Paraffin was removed by xylene extraction followed by washing with ethanol. RNA was isolated from the sectioned tissue blocks using the purification kit, total RNA was extracted, and RNA was subjected to DNase I (Invitrogen, AM2222) treatment. The qRT-PCR was performed using the TransScript II Green One-Step qRT-PCR SuperMix kit (TransGen Biotech, AQ311-01) with 100 ng RNA as template in a 20 μL reaction volume on an ABI 7,500 real-time cycler (Qiagen).

PCR cycling was performed as follows: one cycle at 95°C for 10 min, 95°C for 20 s, and 40 cycles at 60°C for 45 s. The threshold cycle for a given amplification curve during RT-PCR occurs at the point where the fluorescent signal grows beyond a specified fluorescence threshold setting.

The results were normalized with beta actin, and the relative RNA expression was calculated by the 2-ΔΔCt method. To evaluate the statistical significance of PCR data, the paired sample *t*-test was used. The hsa_circ_0003970 primer sequences were as follows: left primer 5′-AGCTGAGGGACAACAACACC-3′; right primer 5′-CCTCTTGTAACCTTTCCTCCA-3′. The hsa_circ_0006410 primer sequences were as follows: left primer 5′-GTGGA ACCATATCCGTGGAC-3′; right primer 5′-GAAAAACGTCT GTCCCCTCA-3′. The hsa_circ_0006754 primer sequences were as follows: left primer 5′-CTGAATTGGAGATGCTTTCAGA-3′; right primer 5′-TGGAGCACAGGTATCAACCA-3′. The hsa_circ_0005848 primer sequences were as follows: left primer 5′-GGGAAGTGCTGGACCTATGA-3′; right primer 5′-TCACGGCTTTTGCTCTTTTT-3′. The hsa_circ_AKAP7 primer sequences were as follows: left primer 5′-AGGCA TCCTGGTAGGAGAGAG-3′; right primer 5′-AGCAAATGG CATGTCTACCA-3′.

## Results

### Differentially Expressed CircRNAs in PCa

**FIGURE 1:**
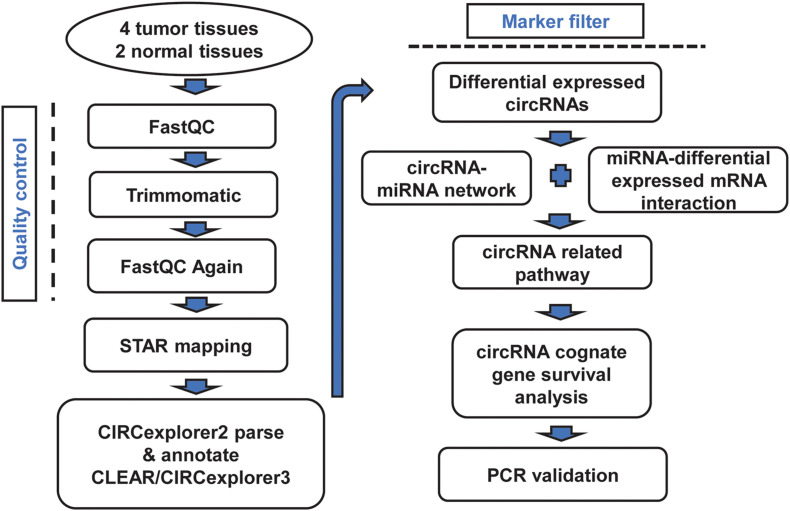
Bioinformatics analysis pipeline of our study.

**FIGURE 2:**
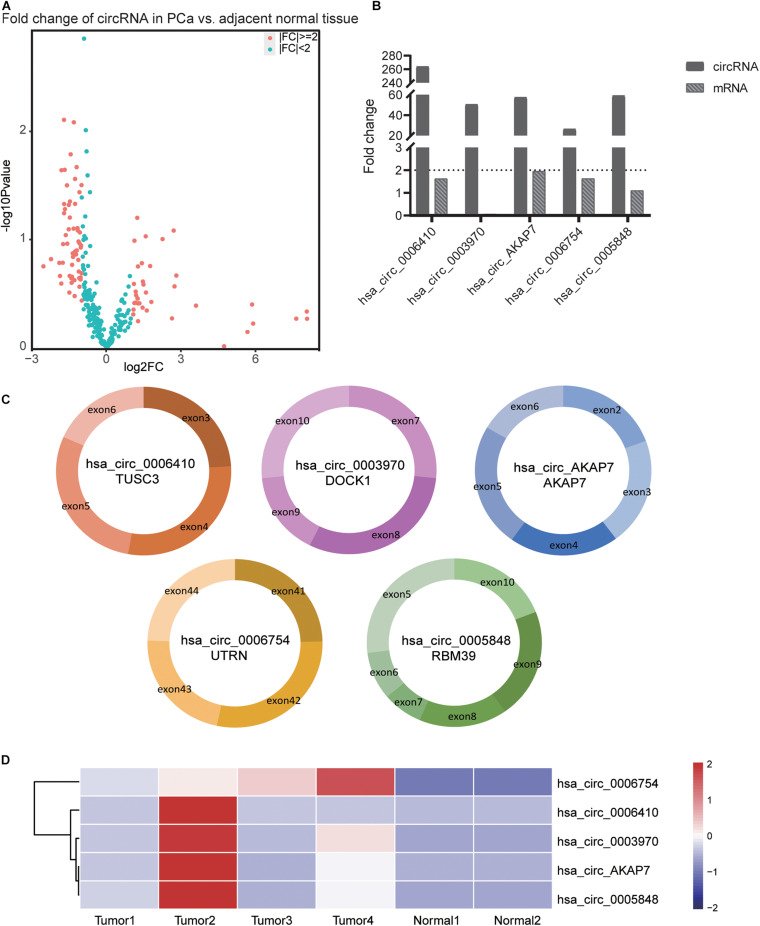
Information of the five PCa specific circRNAs. **(A)** Volcano plot of circRNAs in PCa. The red points represent differentially expressed circRNAs in PCa with fold-change >2 or fold-change <0.5. The paired *t*-test was used to obtain *p*-value. **(B)** The fold-change of circRNAs and its cognate mRNA expression between tumor tissue and adjacent normal tissue. The fold-change of circRNAs was much higher than its mRNAs especially in hsa_circ_0006410. In both tumor as well as normal samples, the expression of hsa_circ_0003970 cognate mRNA was 0. Thus, the mRNA fold change of hsa_circ_0003970 was 0. **(C)** The exon composition of hsa_circ_0006410, hsa_circ_0003970, hsa_circ_0006754, hsa_circ_0005848, and hsa_circ_AKAP7. **(D)** Heatmap of the expression of five specific circRNAs. These five circRNAs are highly expressed in tumor tissues than in normal tissues. The value used in this figure is the expression of circRNAs, and we used “scale = row” to make the graph more consecutive.

Analysis of RNA-seq data (Figure 1) showed 89 differentially expressed circRNAs with fold-change >2 or fold-change <0.5. Due to the small sample size in our sequencing control set, the *p*-value was not accurate for initial screening. The differentially expressed circRNAs included 32 upregulated circRNAs and 57 downregulated circRNAs (Figure 2A). According to this result, we selected the top five differentially expressed circRNAs for further analysis—included hsa_circ_0006410, hsa_circ_0003970, hsa_circ_0006754, hsa_circ_0005848, and a novel circRNA that we named hsa_circ_AKAP7 (Figure 2C and Table 1). Because of the circRNA back-splicing feature, an mRNA may correspond to multiple circRNAs. Figure 2C shows the precise corresponding exon, enabling the identification of its component. These five circRNAs were highly expressed in tumor tissues compared with their cognate mRNAs (Figure 2B). The Pearson correlation coefficients of circRNAs and mRNAs were consistent with those found in previous studies; circRNAs and mRNAs tend to have a high correlation (Yang et al., 2018). The produced circRNAs and their cognate mRNAs usually inhibit each other but Figure 2B did not show an opposite trend of circRNA and mRNA expression. This indicated that these five circRNAs have key functions in tumor tissue instead of their cognate mRNAs. The expression of these five circRNAs is depicted in Figure 2D. The prominent higher expression of these five specific circRNAs led us to further research. Therefore, we regarded circRNAs as miRNA sponges to explore the functions of these five circRNAs.

**TABLE 1:** The detailed information of circRNAs.

**circRNA ID**	**Chrom**	**Start**	**End**	**Strand**	**Cognate mRNA**	**Cognate gene**	**Pearson correlation**
hsa_circ_0006410	chr8	15650696	15673836	+	ENST00000382020.8	TUSC3	0.997861
hsa_circ_0003970	chr10	126996747	127000307	+	ENST00000280333.9	DOCK1	0.962136
hsa_circ_AKAP7	chr6	131145284	131199573	+	ENST00000431975.7	AKAP7	−0.31984
hsa_circ_0006754	chr6	144531051	144539443	+	ENST00000367545.7	UTRN	0.841363
hsa_circ_0005848	chr20	35721739	35732135	−	ENST00000639702.1	RBM39	0.97713

*The genome version is hg38.*

### CircRNA-miRNA Network and miRNA-Related mRNAs

**FIGURE 3:**
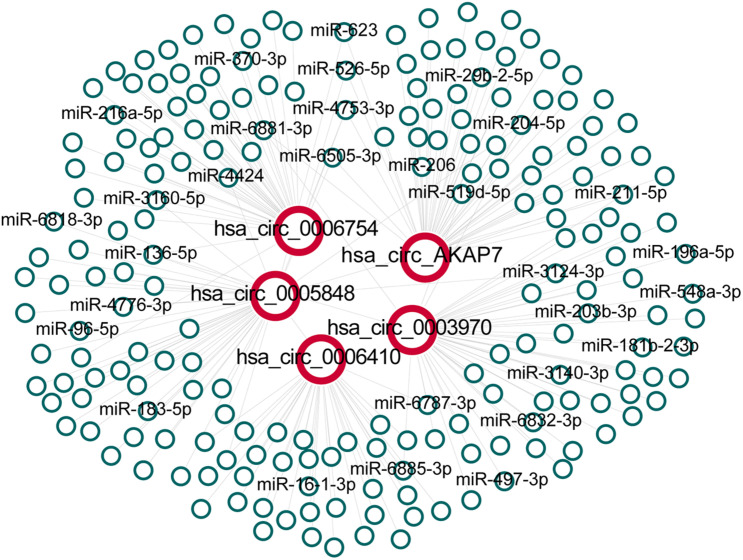
The network between five specific circRNAs and their predicted interactions of miRNAs. All interactions between circRNAs and miRNAs were obtained and extracted for hsa_circ_0006410, hsa_circ_0003970, hsa_circ_0006754, hsa_circ_0005848 and hsa_circ_AKAP7 to build the network.

After obtaining circRNA and miRNA interactions from circBank and miRNDB, we built five circRNA and miRNA interaction networks (Figure 3). We found 215 circRNA-miRNA interactions in the network, and each circRNA had an average of 43 miRNA interactions. Many of these miRNAs have been reported to be associated with PCa (Table 2). Hsa_circ_0003970 and hsa_circ_0005848 interacted with miRNA-204-5p, which is a tumor suppressor that promotes apoptosis by targeting BCL2 in PCa cells (Lin et al., 2017). Hsa_circ_0005848 and hsa_circ_0006754 related miR-3160-5p is a PCa cell proliferation suppressor that targets the F-box protein (Lin et al., 2018). MiR-548 acts as an anti-oncogenic factor that inhibits the phosphoinositide three-kinase (PI3K)/AKT signaling pathway in lung cancer and is associated with high-risk Gleason scores in PCa (Shi et al., 2015). The PI3K/AKT pathway is involved in tumor immunological surveillance and immune suppression (Dituri et al., 2011). Hsa-miR-181b-2-3p and hsa-miR-96-5p were associated with the androgen receptor and Gleason score (Mekhail et al., 2014). These results further highlight the contribution of this study.

**TABLE 2:** The literature mining of circRNAs related miRNAs.

**circRNA ID**	**miRNA ID**	**miRNA description in PCa or other tumors**	**References**
hsa_circ_0003970	hsa-miR-181b-2-3p	AR signaling in PCa; cancer stem cell (CSC) formation in PCa	Mekhail et al., 2014
hsa_circ_0003970	hsa-miR-196a-5p	Associated with SNPs that can be useful in screening for cancer risk	Mekhail et al., 2014
hsa_circ_0003970	hsa-miR-203b-3p	Anti-metastatic in PCa; epithelial to mesenchymal transition (EMT) in PCa	Mekhail et al., 2014
hsa_circ_0003970	hsa-miR-211-5p	Tumor suppressor by targeting ACSL4 in Hepatocellular Carcinoma	Qin et al., 2020
hsa_circ_0003970	hsa-miR-497-3p	Down-regulated in PCa	Mekhail et al., 2014
hsa_circ_0003970	hsa-miR-548a-3p	Anti-oncogenic factor inhibiting the PI3K/AKT signaling pathway in lung cancer and associated with high-risk Gleason scores in prostate cancer	Li et al., 2013
hsa_circ_0003970; hsa_circ_0005848	hsa-miR-204-5p	Tumor suppressor miRNA-204-5p promotes apoptosis by targeting BCL2 in PCa	Mekhail et al., 2014
hsa_circ_0006754	hsa-miR-216a-5p	Inhibits malignant progression in small cell lung cancer: involvement of the Bcl-2 family proteins	Sun et al., 2018
hsa_circ_0006754	hsa-miR-370-3p	Up-regulated in PCa	Mekhail et al., 2014
hsa_circ_0006754; hsa_circ_AKAP7	hsa-miR-526b-5p	hsa_circ_0085539 promotes osteosarcoma progression by regulating miR-526b-5p and SERP1	Mekhail et al., 2014
hsa_circ_0006410	hsa-miR-16-1-3p	Biochemical failure in PCa	Mekhail et al., 2014
hsa_circ_0005848	hsa-miR-183-5p	Up-regulated in PCa	Mekhail et al., 2014
hsa_circ_0005848	hsa-miR-3160-5p	Suppressed prostate cancer cell proliferation	Lin et al., 2018
hsa_circ_0005848	hsa-miR-96-5p	Biochemical failure in PCa;Gleason score in PCa	Mekhail et al., 2014
hsa_circ_0005848; hsa_circ_AKAP7	hsa-miR-623	Suppressed tumor progression in human lung adenocarcinoma	Wei et al., 2016
has_circ_AKAP7	hsa-miR-206	Anti-metastatic in PCa	Mekhail et al., 2014
has_circ_AKAP7	hsa-miR-29b-2-5p	Anti-metastatic in PCa	Mekhail et al., 2014

The miRNA target prediction based on the short seed sequence provided many false positive results. Thus, we filtered differentially expressed mRNAs in PCa to analyze miRNA and mRNA interactions. Only scores >90 interactions were selected to predict circRNAs function. Then, we used functional enrichment analysis to explore the function of the five circRNAs.

### CircRNA-Related Pathways in Metabolism Pathways

**FIGURE 4:**
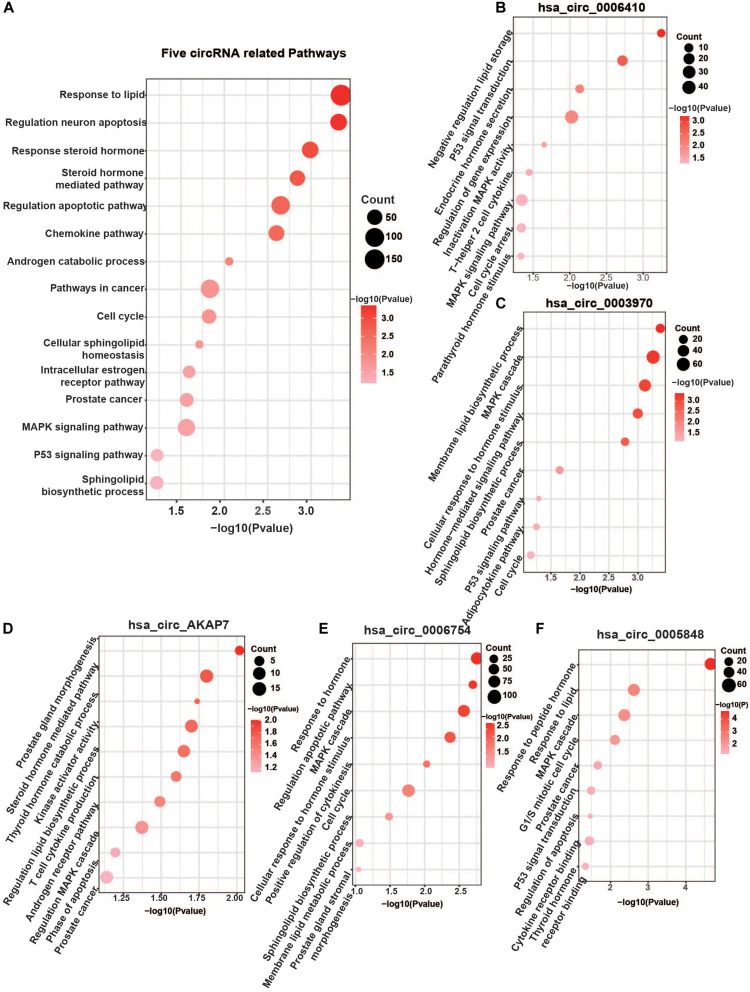
Five specific circRNA-related pathways. **(A)** The five circRNA-related pathways. Only differentially expressed mRNAs with prediction score higher than 90 were considered. Well-known PCa-related pathways, such as the MAPK signaling pathway, P53 pathway, AR pathway, cell cycle, steroid hormone-mediated signaling pathway, and lipid-related process, were all found. **(B)** hsa_circ_0006410 related pathways. **(C)** hsa_circ_0003970 related pathways. **(D)** hsa_circ_AKAP7 related pathways. **(E)** hsa_circ_0006754 related pathways. **(F)** hsa_circ_0005848 related pathways. “Count” represents the number of genes in the relevant categories.

Analysis of these five circRNA-related mRNAs showed that they were all enriched in many well-known PCa pathways (Figure 4A). Hsa_circ_0006410, hsa_circ_0003970, hsa_circ_AKAP7, hsa_circ_0006754, and hsa_circ_0005848 were all related to the MAPK signaling pathway. MAPK signaling is an important regulator of cancer, especially PCa. It includes three cross-signaling pathways: p38, JNK, and ERK (Dhillon et al., 2007). Each pathway comprises several levels of kinases. The p38-MAPK pathway is important for the production of inflammatory cytokines and IFN-γ. It can also positively regulate Th1 differentiation instead of Th2 (Martinez et al., 2009). The JNK–MAPK pathway plays pro-inflammatory roles in macrophages, inducing M1 differentiation. Activation of the ERK–MAPK pathway favors cell differentiation into CD4 lineage and is critical for CD4 T cell polarization of Th2 because it is required for IL-4 receptor function (Alessandro et al., 2019). These regulators are significant in PCa.

Other significant pathways were the hormone-mediated signaling pathway and cellular lipid-related process, which were associated with hsa_circ_0006410, hsa_circ_0003970, hsa_circ_AKAP7, hsa_circ_0006754, and hsa_circ_0005848 (Figures 4B–F). Steroid androgen hormones play key roles in the progression and treatment of PCa. Androgen deprivation therapy (ADT) is the first-line treatment used to control cancer growth (Munkley et al., 2016). It functions by inhibiting the production of male hormone testosterone and preventing it from reaching PCa cells. ADT can cause apoptosis of PCa cells and can make them grow slowly. Studies have indicated that dietary fat intake is related to PCa development, suggesting that lipid metabolism plays a role in the carcinogenesis and progression of PCa (Tamura et al., 2009). Dysregulation of metabolism of lipids, especially sphingolipid, is a hallmark of the malignant phenotype. Increased lipid accumulation leading to changes in levels of lipid metabolic enzymes has been verified in various tumors, including PCa (Wu et al., 2014). Castration-resistant PCa (CRPC) is considered to utilize *de novo* lipid synthesis to produce fatty acids to obtain energy (Eidelman et al., 2017). The five circRNAs were related to both the hormone-mediated signaling pathway and the lipid-related process, indicating that they are involved in PCa regulation. Several pathways found to be closely related to PCa include the chemokine pathway, cell cycle, p53 signaling pathway, apoptosis and transcriptional misregulation in cancer (Figure 4A).

### Survival Analysis of CircRNA Cognate Genes and qRT-PCR Validation of Five CircRNAs

**FIGURE 5:**
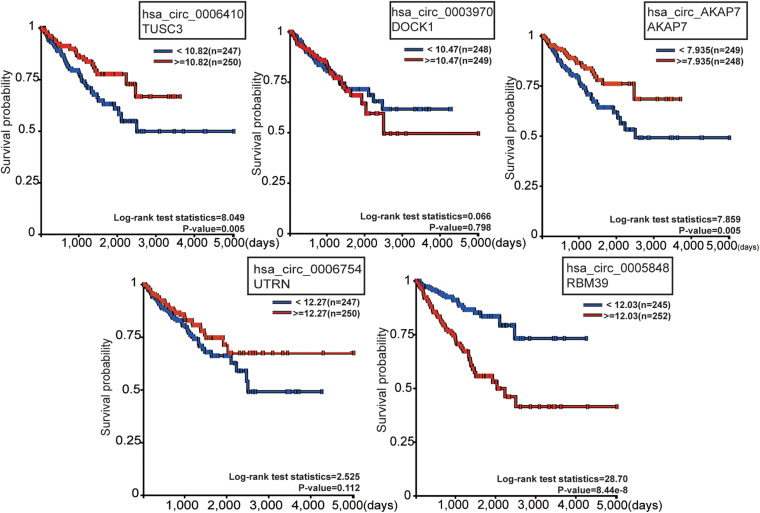
Survival analysis of the five circRNA cognate genes in PCa. Among these, TUSC3, AKAP7, and RBM39 were significantly related with survival probability. The red line represents high gene expression, and the blue line represents low gene expression.

To observe the effect of the five circRNAs in PCa patients, survival analysis of circRNA cognate genes in TCGA data was performed (Figure 5). TUSC3 (hsa_circ_0006410 cognate gene), AKAP7 (hsa_circ_AKAP7 cognate gene), and RBM39 (hsa_circ_0005848 cognate gene) were all significantly associated with progression-free survival of PCa patients, as shown by the Kaplan-Meier plot (*P*-value < 0.05) (Figure 5A). Patients with high expression of TUSC3 and AKAP7 showed better overall survival. This is consistent with the fact that TUSC3 is a tumor suppressor gene (Yu et al., 2017). Meanwhile, low expression of RBM39 was found to be associated with low overall survival. RBM39 is associated with precursor messenger RNA (pre-mRNA) splicing factors, and inactivation of RBM39 causes aberrant pre-mRNA splicing. Previous studies have shown that several single amino acid substitutions in RBM39 confer resistance to the toxic effects of indisulam in cultured cancer cells and in mice with tumor xenografts (Han et al., 2017). Since the direction of differential expression varied among the five mRNAs, we think that circRNAs might act as oncogenes or tumor suppressor genes in PCa. The direction of different functional circRNAs is different in its cognate mRNAs (Figure 5). However, further experiments are required for confirming this.

**FIGURE 6:**
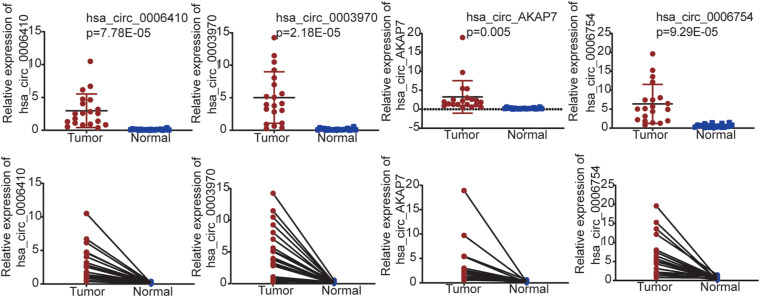
qRT-PCR validation for PCa tissues and adjacent normal tissues in 20 samples from patient samples diagnosed with PCa. Only four circRNAs were significantly validated.

We used qRT-PCR to validate the five circRNAs in 20 PCa and normal samples (Figure 6). In our results, four circRNAs were significantly validated—hsa_circ_0006410, hsa_circ_0003970, hsa_circ_AKAP7, and hsa_circ_0006754. The relative expression of circRNAs indicated that these circRNAs were highly expressed in tumor tissues compared to normal tissues and validated our analysis.

## Discussion

Based on sequencing data of PCa tissues and adjacent normal tissues, we identified differentially expressed circRNAs in PCa. We filtered five specific highly expressed circRNAs that had never been studied in PCa before for further analysis. We found that the miRNAs and mRNA pathways related to these circRNAs were related to known metabolic pathways, such as PI3K-Akt signaling pathway, MAPK signaling pathway, and lipid metabolic process. This also confirmed the reliability of our findings. Through bioinformatics analysis, we analyzed expression levels of circRNA through linear RNA expression level adjustment using CIRCexplorer3. For these five circRNAs, the expression of circRNAs and mRNAs in tumor tissue was highly correlated, which is consistent with the results of previous studies. However, the fold changes of circRNAs expression were notably larger than those of their cognate mRNAs, suggesting that circRNAs play a role in tumor tissues. The underlying mechanism, however, is still unknown and requires further research.

We predicted circRNAs function by using circRNA-miRNA-mRNA interactions and showed that they were all significantly enriched in the lipid metabolism pathway. The link between PCa development and lipid metabolism is well established, with AR intimately involved in a number of lipogenic processes. Altered lipid signatures may offer insights into metabolic reprogramming. Lipid pathway deregulation in advanced PCa is a hot research field to identify a therapeutic pathway. Several therapeutic agents, such as warfarin, atostatin, and orlistat, are known to block key processes in lipid metabolism and negatively influence PCa progression. Lipid metabolism is also activated by the PI3K-Akt signaling pathway by sterol regulatory element-binding protein 1 (SREBP1) (Edlind and Hsieh, 2014). The hsa_circ_0006410, hsa_circ_0003970, hsa_circ_0006754, hsa_circ_0005848, and hsa_circ_AKAP7 were all enriched in lipid related pathways, which shows their potential as targets.

The PI3K-Akt signaling pathway is deregulated in 42% of localized disease and 100% of advanced-stage disease in PCa. This implies that the alteration of this pathway is another factor in the development of CRPC. Gene amplifications, mutations, and changes in mRNA expression of PI3K signaling pathway are highly correlated with PCa patients (Edlind and Hsieh, 2014). Hsa_circ_0006410, hsa_circ_0003970, hsa_circ_0005848, and hsa_circ_AKAP7 were all related with this pathway. CircRNAs have been reported to activate the PI3K/Akt signaling pathway by regulating gene expression in PCa (Wang et al., 2020). Although the exact mechanism affecting PI3K/Akt signaling pathway is unclear, the circRNAs identified in this study also provided support for this field.

We used survival analysis and qRT-PCR to validate our findings. Survival analysis is a good indicator to assess the function of genes. Three cognate genes of these five circRNAs were significantly identified in survival analysis, alluding that their cognate genes were key genes in regulating tumor progression. Meanwhile, four circRNAs were well verified by qRT-PCR, except for hsa_circ_0005848. We inferred that this may be due to the space structure or the false positive expression of hsa_circ_0005848.

Our research was based on the bioinformatics analysis of RNA-seq between prostate tumor tissues and adjacent normal tissues. We found five specific circRNAs that were highly related to the AR signaling pathway, MAPK signaling pathway, hormone-mediated signaling pathway, and cellular lipid metabolic process. Furthermore, survival analysis and qRT-PCR validation also verified that the circRNAs were closely related to tumor progression of PCa. These five circRNAs can provide new solutions for research in this field.

## Data Availability Statement

The datasets presented in this study can be found in online repositories. The names of the repository/repositories and accession number(s) can be found below: Genome Sequence Archive for Human (https://bigd.big.ac.cn/bioproject/browse/PRJCA003890) (accession: PRJCA003890).

## Ethics Statement

The studies involving human participants were reviewed and approved by Beijing Hospital Ethics Committee. The patients/participants provided their written informed consent to participate in this study. Written informed consent was obtained from the individual(s) for the publication of any potentially identifiable images or data included in this article.

## Author Contributions

LZ and WZ conceived the study and wrote the manuscript. LZ, FS, and YZ designed the detail analysis pipeline. LZ and FS did the bioinformatics analysis. WZ, HL, XT, and SX performed the experiments. MW and LW participated in revising the manuscript. All authors read and approved the final manuscript.

## Conflict of Interest

The authors declare that the research was conducted in the absence of any commercial or financial relationships that could be construed as a potential conflict of interest.

## References

[B1] Alessandro M. S. Golombek D. A. Chiesa J. J. (2019). Protein kinases in the photic signaling of the mammalian circadian clock. *Yale J. Biol. Med*. 92 241–250. 31249485 PMC6585524

[B2] Andrews S. (2010). *Fastqc**: A Quality Control Tool for High Throughput Sequence Data.* Available online at: https://www.bioinformatics.babraham.ac.uk/projects/fastqc/ (accessed August 10, 2020).

[B3] Arnaiz E. Sole C. Manterola L. Iparraguirre L. Otaegui D. Lawrie C. H. (2019). CircRNAs and cancer: biomarkers and master regulators. *Semin. Cancer Biol*. 58 90–99. 10.1016/j.semcancer.2018.12.002 30550956

[B4] Bolger A. M. Lohse M. Usadel B. (2014). Trimmomatic: a flexible trimmer for illumina sequence data. *Bioinformatics* 30 2114–2120. 10.1093/bioinformatics/btu170 24695404 PMC4103590

[B5] Carroll P. H. Mohler J. L. (2018). NCCN guidelines updates: prostate cancer and prostate cancer early detection. *J. Natl. Compr. Canc. Netw*. 16 620–623. 10.6004/jnccn.2018.0036 29784740

[B6] Chen Y. Wang X. (2020). miRDB: an online database for prediction of functional microRNA targets. *Nucleic Acids Res*. 48 D127–D131. 10.1093/nar/gkz757 31504780 PMC6943051

[B7] Deng Z. Li X. Wang H. Geng Y. Cai Y. Tang Y. (2020). Dysregulation of circRNA_0001946 contributes to the proliferation and metastasis of colorectal cancer cells by targeting microRNA-135a-5p. *Front. Genet*. 11:357 10.3389/fgene.2020.00357 PMC7232565 32508871

[B8] Dhillon A. S. Hagan S. Rath O. Kolch W. (2007). MAP kinase signalling pathways in cancer. *Oncogene* 26 3279–3290. 10.1038/sj.onc.1210421 17496922

[B9] Dituri F. Mazzocca A. Giannelli G. Antonaci S. (2011). PI3K functions in cancer progression, anticancer immunity and immune evasion by tumors. *Clin. Dev. Immunol*. 2011:947858 10.1155/2011/947858 PMC3199188 22046194

[B10] Dobin A. Davis C. A. Schlesinger F. Drenkow J. Zaleski C. Jha S. (2013). STAR: ultrafast universal RNA-seq aligner. *Bioinformatics* 29 15–21. 10.1093/bioinformatics/bts635 23104886 PMC3530905

[B11] Edlind M. P. Hsieh A. C. (2014). PI3K-AKT-mTOR signaling in prostate cancer progression and androgen deprivation therapy resistance. *Asian J. Androl*. 16 378–386. 10.4103/1008-682X.122876 24759575 PMC4023363

[B12] Eidelman E. Twum-Ampofo J. Ansari J. Siddiqui M. M. (2017). The metabolic phenotype of prostate cancer. *Front. Oncol*. 7:131 10.3389/fonc.2017.00131 PMC5474672 28674679

[B13] Etzioni R. Nyame Y. A. (2020). Prostate cancer screening guidelines for Black men: spotlight on an empty stage. *J. Natl. Cancer Inst*. djaa172 10.1093/jnci/djaa172 PMC8168118 33146382

[B14] Feng Y. Yang Y. Zhao X. Fan Y. Zhou L. Rong J. (2019). Circular RNA circ0005276 promotes the proliferation and migration of prostate cancer cells by interacting with FUS to transcriptionally activate XIAP. *Cell Death Dis*. 10:792 10.1038/s41419-019-2028-9 PMC6797747 31624242

[B15] Goldman M. J. Craft B. Hastie M. Repecka K. McDade F. Kamath A. (2020). Visualizing and interpreting cancer genomics data via the Xena platform. *Nat. Biotechnol*. 38 675–678. 10.1038/s41587-020-0546-8 32444850 PMC7386072

[B16] Han T. Goralski M. Gaskill N. Capota E. Kim J. Ting T. C. (2017). Anticancer sulfonamides target splicing by inducing RBM39 degradation via recruitment to DCAF15. *Science* 356:eaal3755 10.1126/science.aal3755 28302793

[B17] Hansen T. B. Jensen T. I. Clausen B. H. Bramsen J. B. Finsen B. Damgaard C. K. (2013). Natural RNA circles function as efficient microRNA sponges. *Nature* 495 384–388. 10.1038/nature11993 23446346

[B18] Jeck W. R. Sharpless N. E. (2014). Detecting and characterizing circular RNAs. *Nat. Biotechnol*. 32 453–461. 10.1038/nbt.2890 24811520 PMC4121655

[B19] Jeck W. R. Sorrentino J. A. Wang K. Slevin M. K. Burd C. E. Liu J. (2013). Circular RNAs are abundant, conserved, and associated with ALU repeats. *RNA* 19 141–157. 10.1261/rna.035667.112 23249747 PMC3543092

[B20] Kitagawa Y. Ueno S. Izumi K. Kadono Y. Konaka H. Mizokami A. (2014). Cumulative probability of prostate cancer detection in biopsy according to free/total PSA ratio in men with total PSA levels of 2.1-10.0 ng/ml at population screening. *J. Cancer Res. Clin. Oncol*. 140 53–59. 10.1007/s00432-013-1543-9 24165867 PMC11824128

[B21] Lander E. S. Linton L. M. Birren B. Nusbaum C. Zody M. C. Baldwin J. (2001). Initial sequencing and analysis of the human genome. *Nature* 409 860–921. 10.1038/35057062 11237011

[B22] Li S. Yu C. Zhang Y. Liu J. Jia Y. Sun F. (2020). Circular RNA cir-ITCH is a potential therapeutic target for the treatment of castration-resistant prostate cancer. *Biomed. Res. Int*. 2020:7586521 10.1155/2020/7586521 PMC7456474 32904490

[B23] Li Y. Xie J. Xu X. Wang J. Ao F. Wan Y. (2013). MicroRNA-548 down-regulates host antiviral response via direct targeting of IFN-lambda1. *Protein Cell* 4 130–141. 10.1007/s13238-012-2081-y 23150165 PMC4875363

[B24] Liao Y. Smyth G. K. Shi W. (2014). featureCounts: an efficient general purpose program for assigning sequence reads to genomic features. *Bioinformatics* 30 923–930. 10.1093/bioinformatics/btt656 24227677

[B25] Lin P. Zhu L. Sun W. Yang Z. Sun H. Li D. (2018). Prostate cancer cell proliferation is suppressed by microRNA-3160-5p via targeting of F-box and WD repeat domain containing 8. *Oncol. Lett*. 15 9436–9442. 10.3892/ol.2018.8505 29805667 PMC5958718

[B26] Lin Y. C. Lin J. F. Tsai T. F. Chou K. Y. Chen H. E. Hwang T. I. (2017). Tumor suppressor miRNA-204-5p promotes apoptosis by targeting BCL2 in prostate cancer cells. *Asian J. Surg*. 40 396–406. 10.1016/j.asjsur.2016.07.001 27519795

[B27] Litwin M. S. Tan H. J. (2017). The diagnosis and treatment of prostate cancer: a review. *JAMA* 317 2532–2542. 10.1001/jama.2017.7248 28655021

[B28] Liu M. Wang Q. Shen J. Yang B. B. Ding X. (2019). Circbank: a comprehensive database for circRNA with standard nomenclature. *RNA Biol*. 16 899–905. 10.1080/15476286.2019.1600395 31023147 PMC6546381

[B29] Ma X. K. Wang M. R. Liu C. X. Dong R. Carmichael G. G. Chen L. L. (2019). CIRCexplorer3: a Clear pipeline for direct comparison of circular and linear RNA expression. *Genom. Proteom. Bioinform*. 17 511–521. 10.1016/j.gpb.2019.11.004 PMC7056929 31904419

[B30] Martinez F. O. Helming L. Gordon S. (2009). Alternative activation of macrophages: an immunologic functional perspective. *Annu. Rev. Immunol*. 27 451–483. 10.1146/annurev.immunol.021908.132532 19105661

[B31] Mekhail S. M. Yousef P. G. Jackinsky S. W. Pasic M. Yousef G. M. (2014). miRNA in prostate cancer: new prospects for old challenges. *EJIFCC* 25 79–98. 27683458 PMC4975192

[B32] Munkley J. Vodak D. Livermore K. E. James K. Wilson B. T. Knight B. (2016). Glycosylation is an androgen-regulated process essential for prostate cancer cell viability. *EBioMedicine* 8 103–116. 10.1016/j.ebiom.2016.04.018 27428423 PMC4919605

[B33] PCa (2019). NICE guidance – prostate cancer: diagnosis and management: (c) NICE (2019) Prostate cancer: diagnosis and management. *BJU Int*. 124 9–26. 10.1111/bju.14809 31206997

[B34] Qin X. Zhang J. Lin Y. Sun X. M. Zhang J. N. Cheng Z. Q. (2020). Identification of MiR-211-5p as a tumor suppressor by targeting ACSL4 in *Hepatocellular carcinoma*. *J. Transl. Med*. 18:326 10.1186/s12967-020-02494-7 PMC7456023 32859232

[B35] Robinson M. D. McCarthy D. J. Smyth G. K. (2010). edgeR: a Bioconductor package for differential expression analysis of digital gene expression data. *Bioinformatics* 26 139–140. 10.1093/bioinformatics/btp616 19910308 PMC2796818

[B36] Salzman J. Gawad C. Wang P. L. Lacayo N. Brown P. O. (2012). Circular RNAs are the predominant transcript isoform from hundreds of human genes in diverse cell types. *PLoS One* 7:e30733 10.1371/journal.pone.0030733 PMC3270023 22319583

[B37] Shannon P. Markiel A. Ozier O. Baliga N. S. Wang J. T. Ramage D. (2003). Cytoscape: a software environment for integrated models of biomolecular interaction networks. *Genome Res*. 13 2498–2504. 10.1101/gr.1239303 14597658 PMC403769

[B38] Shi Y. Qiu M. Wu Y. Hai L. (2015). MiR-548-3p functions as an anti-oncogenic regulator in breast cancer. *Biomed. Pharmacother*. 75 111–116. 10.1016/j.biopha.2015.07.027 26297544

[B39] Siegel R. L. Miller K. D. Jemal A. (2017). Cancer statistics, 2017. *CA Cancer J. Clin*. 67 7–30. 10.3322/caac.21387 28055103

[B40] Sun Y. Hu B. Wang Y. Li Z. Wu J. Yang Y. (2018). miR-216a-5p inhibits malignant progression in small cell lung cancer: involvement of the Bcl-2 family proteins. *Cancer Manag. Res*. 10 4735–4745. 10.2147/CMAR.S178380 30425570 PMC6201844

[B41] Suzuki H. Zuo Y. Wang J. Zhang M. Q. Malhotra A. Mayeda A. (2006). Characterization of RNase R-digested cellular RNA source that consists of lariat and circular RNAs from pre-mRNA splicing. *Nucleic Acids Res*. 34:e63 10.1093/nar/gkl151 PMC1458517 16682442

[B42] Tamura K. Makino A. Hullin-Matsuda F. Kobayashi T. Furihata M. Chung S. (2009). Novel lipogenic enzyme ELOVL7 is involved in prostate cancer growth through saturated long-chain fatty acid metabolism. *Cancer Res*. 69 8133–8140. 10.1158/0008-5472.CAN-09-0775 19826053

[B43] Tay Y. Rinn J. Pandolfi P. P. (2014). The multilayered complexity of ceRNA crosstalk and competition. *Nature* 505 344–352. 10.1038/nature12986 24429633 PMC4113481

[B44] Wang Y. Yin L. Sun X. (2020). CircRNA hsa_circ_0002577 accelerates endometrial cancer progression through activating IGF1R/PI3K/Akt pathway. *J. Exp. Clin. Cancer Res*. 39:169 10.1186/s13046-020-01679-8 PMC7450704 32847606

[B45] Wei S. Zhang Z. Y. Fu S. L. Xie J. G. Liu X. S. Xu Y. J. (2016). Hsa-miR-623 suppresses tumor progression in human lung adenocarcinoma. *Cell Death Dis*. 7:e2388 10.1038/cddis.2016.260 PMC5059863 27685632

[B46] Wickham H. (2016). *ggplot2: Elegant Graphics for Data Analysis.* New York, NY: Springer-Verlag.

[B47] Wu X. Daniels G. Lee P. Monaco M. E. (2014). Lipid metabolism in prostate cancer. *Am. J. Clin. Exp. Urol*. 2 111–120. 25374912 PMC4219300

[B48] Yang Q. Wu J. Zhao J. Xu T. Zhao Z. Song X. (2018). Circular RNA expression profiles during the differentiation of mouse neural stem cells. *BMC Syst. Biol*. 12(Suppl. 8):128 10.1186/s12918-018-0651-1 PMC6302452 30577840

[B49] Young M. D. Wakefield M. J. Smyth G. K. Oshlack A. (2010). Gene ontology analysis for RNA-seq: accounting for selection bias. *Genome Biol*. 11:R14 10.1186/gb-2010-11-2-r14 PMC2872874 20132535

[B50] Yu T. Wang Y. Fan Y. Fang N. Wang T. Xu T. (2019). CircRNAs in cancer metabolism: a review. *J. Hematol. Oncol*. 12:90 10.1186/s13045-019-0776-8 PMC6727394 31484561

[B51] Yu X. Zhai C. Fan Y. Zhang J. Liang N. Liu F. (2017). TUSC3: a novel tumour suppressor gene and its functional implications. *J. Cell. Mol. Med*. 21 1711–1718. 10.1111/jcmm.13128 28272772 PMC5571513

[B52] Zhang X. O. Dong R. Zhang Y. Zhang J. L. Luo Z. Zhang J. (2016). Diverse alternative back-splicing and alternative splicing landscape of circular RNAs. *Genome Res*. 26 1277–1287. 10.1101/gr.202895.115 27365365 PMC5052039

